# Psychometric evaluation of the Chinese version of the Multidimensional Competitive Orientation Inventory

**DOI:** 10.1038/s41598-024-57359-6

**Published:** 2024-03-19

**Authors:** Yuqian Wang, Gabor Orosz, Xi Chen, Chengguo Miao, Yansong Li

**Affiliations:** 1 Reward, Competition, and Social Neuroscience Lab, Department of Psychology, School of Social and Behavioral Sciences, Nanjing University, Nanjing, 210023 China; 2 Unité de Recherche Pluridisciplinaire Sport Santé Société, Université d’Artois, Sherpas, Liévin, France; 3 Institute for Brain Sciences, Nanjing University, Nanjing, China

**Keywords:** Competition, MCOI, Chinese, Culture, HCO, SDCO, ADCO, LIC, Psychology, Human behaviour

## Abstract

This study examined the psychometric properties of the Chinese version of the Multidimensional Competitive Orientation Inventory (Ch-MCOI) in adults from Mainland China. A total of 1121 participants (50.6% male; M = 28.86, SD = 8.70) were recruited for this study. All participants completed the Chinese versions of the MCOI, the Connor-Davidson Resilience Scale (CD-RISC), the Warwick-Edinburgh Mental Well-being Scale (WEMWBS), the Almost Perfect Scale-Revised (APS), the Frost Multidimensional Perfectionism Scale (MPS-f), and the Competition Attitude Scale (Ch-CAS). A subsample of 239 participants (50.6% male; M = 32.04, SD = 8.13) completed the Ch-MCOI again after a two-week interval to assess test–retest reliability. Exploratory Structural Equation Modeling (ESEM) yielded a four-factor structure (hyper-competitive orientation, self-developmental competitive orientation, anxiety-driven competition avoidance, and lack of interest toward competition), which was further validated by confirmatory factor analyses with a satisfactory fit. Furthermore, test–retest reliability, internal consistency, and convergent and concurrent validity were also acceptable. Our findings suggest that the Ch-MCOI could be a reliable and valid instrument for assessing the adaptive and maladaptive facets of competitive orientations in the Chinese-speaking population.

## Introduction

From the seminal work of Horney^1^, through the groundbreaking experiments and theory of^2,3^, up to more recent research, individual differences in the competition have been at the center of popular and scientific psychological inquiry (see also^4,5^). Assessing modern forms of individual differences in competition reaches back to the pioneers in the field. In 1937, Horney described the maladaptive aspect of competition as neurotic competitiveness including a combination of hyper-competitiveness and competition avoidance. In her interpretation, hyper-competition was one kind of neurotic competition where individuals want to surpass others at any cost and in any situation^1^. It is related to the cultural belief in the U.S. that winning is of great importance^6–8^. According to Horney^1^, the other maladaptive aspect of neurotic competitive behavior is competition avoidance. In this orientation, people believe that they will be rejected as a result of their failures; as such, they are nervous and stressed about potential competitive failures. From the perspective of individual differences, competition has adaptive aspects, such as personal-development competitive attitudes^9^. The personal-development competitive orientation focuses on personal development, growth, and the enjoyment of competitive tasks with elevated psychological health risks and consideration of the well-being of others^9^.

One of the new approaches to competitive attitudes aimed to demonstrate that personal orientation towards competition is not dichotomous (e.g., adaptive vs. maladaptive), it involves multiple aspects. This calls for efforts to develop a reliable inventory measuring distinct aspects of competitive orientations. The Multidimensional Competitive Orientation Inventory (MCOI) developed by Orosz et al.^10^ can be viewed as an important advance in this field. It is a 12-item scale with excellent or good quality for any of the psychometric properties in a Hungarian non-clinical community sample. Although it is a brief inventory, it allows for measuring four distinct aspects of competitive orientations: Hyper-Competitive Orientation (HCO), Self-Developmental Competitive Orientation (SDCO), Anxiety-Driven Competition Avoidance (ADCA), and Lack of Interest in Competition (LIC). Hyper-Competitive Orientation (HCO) is based on Ryckman et al.’s^11^ concept as it is related to a strong focus on results where the end justifies the means. Self-Developmental Competitive Orientation (SDCO) is also similar to Ryckman, et al.^9^ construct as it emphasizes the individual's own development and ability enhancement. Anxiety-Driven Competition Avoidance (ADCA) is related to Ryckman et al.'s competition avoidance^12^ dimension as it focuses on anxiety rather than the fear of losing the approval of others. Finally, the fourth factor, Lack of Interest in Competition (LIC), is related to the lack of motivation or disinterest in participating in competitive situations. This MCOI seems to provide a reliable and valid instrument for assessing the adaptive and maladaptive facets of competitive orientations. However, its psychometric properties were only examined in the Hungarian sample. This highlights a need to test its universality across cultures and languages. Currently, the psychometric properties of the Hungarian version of the MCOI have been evaluated in the French-speaking population^13^. The French version of the MCOI showed appropriate internal consistency, convergent and concurrent validity, and test–retest reliability, which were comparable to those of the Hungarian version. In this case, the French version of the MCOI will be useful to assess competitive orientations in French-speaking countries. Notably, both Hungarian and French versions are underpinned by a European cultural framework. This commonality in the cultural background did not necessitate an exploration into the nuanced interplay between competitive orientations and cultural contexts. However, the adaptation of this scale into the Chinese context introduces a divergent cultural backdrop. The cultural influence underscores the necessity of a thorough examination of the impact of distinct cultural dimensions on competitive orientations, thereby enriching the scale’s applicability and interpretative validity across cultures. This poses the question of whether these competitive orientations appear and can be distinguished in Chinese culture and whether the MCOI is mainly of use in Western cultural contexts.

Prior research has begun to identify cultural differences, particularly regarding collective cultures, in both competitive situations and competitive orientations^14,15^. For example, the competitive-cooperative dimension is one of the pivotal characteristics that can distinguish between collectivist and individualist cultural contexts. Triandis^16^ defined collectivism as a strong willingness to cooperate with in-group members, rather than drawing personal attention to themselves. From this perspective, China is a collectivist country, strongly influenced by Taoism and Confucianism which encourage interpersonal harmony^17^.

One of the key concepts of Confucianism is *Junzi*^18^ which is not only an abstract historical and religious phenomenon but a construct that is used in contemporary scientific and everyday discourse. *Junzi* was translated into English as “gentleman”, but its actual meaning is much richer^19^. Confucianism defines *Junzi* as an ideal personality that has multiple subdimensions. For instance, Ge et al.^20^, based on the Confucian traditions, assessed *Junzi* based on five dimensions: (1) “wisdom, benevolence, and courage”; (2) “respectfulness and propriety”; (3) “self-cultivation rather than contentions with others”; (4) “conversancy with righteousness and cherished of benign rule”; and (5) “refraining from what should not be done”. These dimensions of *Junzi* are strongly and negatively related to the HCO dimension^20^. Another core concept of Confucianism is humility, which implies that competition with others is immoral^21^. This is in line with prior work demonstrating that people with a collectivist cultural background experience guilt or anxiety when competing with others^22^. Based on these values and empirical results, it can be expected that the ADCA dimension will be relevant in the Chinese cultural context. Based on these cultural traditions, one can expect that Chinese people will disapprove of maladaptive forms of competition (ADCA) or they will be not interested in competitive activities. In the past decades, however, as a result of the expansion of the socialist market economy and the burgeoning of the population, Chinese youth face tremendous competition today. Two prominent Chinese competition-related notions have become salient in everyday culture *Neijuan* (translated as “Involution”) and *Tangping* (translated as “Lying Flat”)^23^. *Neijuan* characterizes “irrational internal competitiveness, or 'voluntary' competition”^24^. This relates to changes when a pattern reaches a specific structure and is incapable of stabilizing or converting into a new form; instead, the pattern continues to complexify. In this high-intensity competitive process in which there is no stabilizing point but increasing levels of complexity, we can identify certain hyper-competitive elements. As an opposing concept, *Tangpin* is synonymous with giving up or losing interest in competition. In short, Chinese youth can be simultaneously influenced by traditional and more recent cultural values and societal norms. Taken together with these contextual elements, the four MCOI dimensions identified in prior work could be relevant in the Chinese cultural context.

The Chinese versions of scales measuring individual differences in competition available date back to earlier work by Chen et al.^21^. They present the Chinese version of the Competitive Attitude Scale (Ch-CAS), which includes the Hyper-competitive Attitude subscale and the Personal Development Competitive Attitude subscale. Since this early work, Xie et al.^25^ developed a Chinese version of the Cooperative and Competitive Personality Scale (Ch-CCPS), which consists of three factors: feelings for competition, beliefs about competition, and behavioral tendencies of competition. The measurement of the maladaptive aspects of competition was not included in the scales described above. As such, there is a major need to develop a scale that can assess both adaptive and maladaptive aspects of competition with good psychometric standards in Chinese samples. To address this issue, this study assessed the psychometric properties of the MCOI on Chinese samples (N = 1121, 50.6% male, M = 28.86, SD = 8.70) and examined its factor structure, reliability, convergent and concurrent validity. Given that there is increasing literature on gender differences in competition^26^, we also attempted to explore the role of gender in the Ch-MCOI.

## Methods

### Participants

We assessed 1121 participants (50.6% male, M = 28.86, SD = 8.70). Data was collected using a third-party platform (Wenjuanxing (WJX) Platform), which is a Chinese online marketplace. All participants completed an online survey containing the measurements listed below in return for 10 RMB (about $1.45). Data quality was controlled through system control and manual checking. In the system control, the WJX platform excluded respondents with the same IP, the same device, or an incorrect answer to lie questions (e.g., What is the capital of China?). In manual checking, WJX staff excluded respondents with an abnormal completion time (i.e., extremely long or short), inconsistent responses (e.g., conflicting responses to two items with the same content), or inattentive responses (e.g. all responses identical on a measurement). Thus, our final sample size following data quality control was 1121 participants. Moreover, a sample of 239 participants (50.6% male, M = 32.04, SD = 8.13) completed the Ch-MCOI again after a two-week interval to assess test–retest reliability. All participants gave written informed consent prior to participation. This study was approved by the Ethical Review Board of the University of Nanjing. This study has been performed in accordance with the ethical standards laid down in the 1964 Declaration of Helsinki and its later amendments.

### Measures

#### The Chinese version of the Multidimensional Competitive Orientation Inventory (Ch-MCOI)

The MCOI is a 12-item self-report instrument that measures four facets of competitive orientation. Each item was rated on a Likert-6 point scale from 1 (not true to me at all) to 6 (completely true to me)^27^. It consists of the following four subscales: Hyper-Competitive Orientation, Self-Developmental Competitive Orientation, Anxiety-Driven Competition Avoidance, and Lack of Interest in Competition. As suggested by Ember and Ember^26^ and Beaton et al.^28^, a two-way translation was performed independently. Specifically, two bilingual translators in China first translated the items of the MCOI into Chinese. Then, two bilingual translators in the U.S.A. back-translated their work. The original and back-translated versions were compared and discussed by the authors and translators. The purpose of this work was to minimize discrepancies between the translated instrument and the original instrument.

### The Chinese version of the Connor-Davidson Resilience Scale (Ch-CD-RISC)

The Chinese version of the CD-RISC is a 10-item self-report instrument that assesses the level of individual psychological resilience^29^. Each item was rated on a Likert-5 point scale from 0 (not true at all) to 4 (true nearly all the time). The higher the score, the higher the perceived resilience level; the lower the score, the lower the perceived resilience level. Cronbach’s α was 0.91 in this study, demonstrating adequate reliability.

### The Chinese version of the Warwick-Edinburgh Mental Well-being Scale (Ch-WEMWBS)

The Chinese version of the WEMWBS is a 14-item self-report instrument that assesses the quality of life in terms of mental and social well-being^30^. Each item is rated on a Likert-5 point scale from 1 (none of the time) to 5 (all of the time). The higher the score, the higher the perceived level of quality of life; the lower the score, the lower the perceived level of quality of life. Cronbach’s α was 0.94 in this study, demonstrating adequate reliability.

### The Chinese version of the Almost Perfect Scale-Revise (Ch-APS-Revised)

The Chinese version of the APS-Revised is used to assess positive and negative aspects of perfectionism and refers to another 22-item self-report instrument^31^. Each item is rated on a Likert-7 point scale from 1 (strongly disagree) to 7 (strongly agree). It contains the following three subscales: high standards, order, and discrepancy. Cronbach’s α for the total scale was 0.91 in this study, demonstrating adequate reliability.

### The Chinese version of the Frost Multidimensional Perfectionism Scale (Ch-MPS-f)

Multidimensional perfectionism was measured using the Chinese version of the MPS-f^32^, which comprises 27 items, rated on a Likert-5 point scale ranging from 1 (Strongly disagree) to 5 (Strongly agree). It consists of the following 5 subscales: Concern over Mistakes, Doubt about Action, Parental Expectation, Organization, and Personal Standard. Cronbach’s α for the total scale was 0.89 in this study, demonstrating adequate reliability.

### The Chinese version of the Competition Attitude Scale (Ch-CAS)

The Chinese version of the Competition Attitude Scale (Ch-CAS) includes 27 items, rated on a Likert-5 point scale ranging from 1 (Strongly disagree) to 5 (Strongly agree)^21^. It consists of a benign competitive attitude subscale emphasizing individuals’ attitude towards competition in terms of individuals’ own development and ability enhancement and a negative competitive attitude subscale emphasizing individuals’ attitude towards competition in terms of competing and winning at all costs as a means of maintaining or enhancing one's self-worth. This scale showed a Cronbach’s alpha of 0.92, indicating high reliability.

### Statistical analysis

Statistical analysis was involved in the following steps: (1) descriptive statistics calculations, (2) item analysis, (3) Exploratory Structural Equation Modeling (ESEM) and confirmatory factor analysis (CFA), and (4) assessment of reliability and, convergent and concurrent validity.

Specifically, calculations were made to identify descriptive statistics for the separate items of the Ch-MCOI for all the valid data including means, standard deviations, skewness, and kurtosis. An acceptable range indicating the normality of the items (skewness within ± 2.00 and kurtosis within ± 7.00) was adopted^33,34^. The data was split into two subsamples via odd and even numbers, with one serving as an exploratory subsample (Subsample 1, n = 560) and the other serving as a validation subsample (Subsample 2, n = 561). The item analysis of the Ch-MCOI was performed on Subsample 1 and determined the validity of each item using two methods. As the original English version of the MCOI is a multidimensional scale, the first method involved calculating the item-total correlation (ITC) between the score of each item of a factor of the Ch-MCOI and the total score for that factor. The same procedure for performing item analysis as that described in the English version of the MCOI^27^ was used for this. The second method involved the use of the critical ratio (CR) to determine the level of item discrimination for each factor of the Ch-MCOI. Then, to reveal the factor structures of the Ch-MCOI, we employed the same method as that described in the English version of the MCOI^27^. We conducted Exploratory Structural Equation Modeling (ESEM) on Subsample 1 and Subsample 2, to examine and validate the proposed four-factor structure of all 12 items. ESEM is a method of combining Exploratory Factor Analysis (EFA) and Confirmatory Factor Analysis (CFA)^35,36^ which enables the explicit description of item-level cross-loadings and also makes it feasible to fix these cross-loadings to zero^37^. The following seven fit indices were reviewed to assess the goodness-of-fit of the proposed four-factor structure of the Ch-MCOI, including the Chi-square statistic, root mean square error of approximation (RMSEA), standardized root mean square residual (SRMR), Bentler– Bonett normed fit index (NFI), incremental fit index (IFI), comparative fit index (CFI), and the Tucker-Lewis Index (TLI). Based on the recommendations by previous research^38,39^, 0.90 is thought to indicate a good fit for the NFI, IFI, CFI, and TLI indices. For the RMSEA and SRMR, values below 0.05 indicate a good fit, and values between 0.05 and 0.08 indicate an acceptable fit. In addition, reliability was assessed in terms of internal consistency reliability and test–retest reliability. Both Cronbach’s alpha (α) and composite reliability (CR) were used to measure the internal consistency. Given that the original version of the MCOI is a multidimensional scale, Cronbach’s alpha (α) and composite reliability (CR) were calculated for each factor of the Ch-MCOI, with values of 0.70 or higher indicating good reliability^40,41^. The 2-week test–retest reliability was also assessed for each factor of the Ch-MCOI via intra-class correlations (ICC). Meanwhile, both the convergent and concurrent validity of the Ch-MCOI were assessed. The average variance extracted (AVE) was estimated for each factor of the Ch-MCOI, with values of 0.50 or higher confirming the convergent validity. We followed the same procedure for assessing the concurrent validity of the English version of the MCOI^27^. Specifically, the concurrent validity of the Ch-MCOI was estimated through partial correlations to examine the relationship between the scores of each facet of the Ch-MCOI and the scores of the Ch-CD-RISC, the Ch-WEMWBS, the Ch-APS-Revised, the Ch-MPS-f, and Ch-CAS. Finally, given that gender differences in competition have been reported^26,42,43^, we further explored whether there is a potential gender effect on the Ch-MCOI scores using an independent sample t-test. Before doing this, we evaluated the invariance across gender groups for equality of the overall factor structure (configural invariance), equality of item factor loadings (metric invariance), and equality of item intercepts (scalar invariance)^44,45^.

All data analyses were performed using IBM SPSS Statistics 25. ESEM was conducted with Mplus 7.4. A *p*-value of < 0.05 was considered to indicate a statistically significant finding.

## Results

### Descriptive statistics

The descriptive statistics for each item are provided in Table 1, including means, standard deviations, skewness, and kurtosis. All skewness and kurtosis statistics were within the acceptable range (skewness within ± 2.00 and kurtosis within ± 7.00), which indicates that item scores followed an approximately normal distribution.

**Table 1:** Each item of the Chinese version of the Multidimensional Competitive Orientation Inventory—descriptive statistics.

Item	*M*	*SD*	Skewness	Kurtosis
Item1	3.20	1.28	0.10	*−*0.68
Item2	3.48	1.29	−0.13	*−*0.62
Item3	2.93	1.32	0.41	*−*0.61
Item4	3.24	1.30	0.18	*−*0.63
Item5	3.04	1.34	0.26	*−*0.66
Item6	2.12	1.31	1.16	0.54
Item7	3.35	1.28	*−*0.11	*−*0.68
Item8	4.16	1.17	*−*0.74	0.42
Item9	3.36	1.32	*−*0.02	*−*0.77
Item10	3.77	1.19	*−*0.33	*−*0.10
Item11	3.71	1.28	*−*0.25	*−*0.45
Item12	3.79	1.25	*−*0.19	*−*0.44

*M* mean; *SD* standard deviation.

### Item analysis

Our item-total correlation (ITC) test showed that the correlation coefficient (r) was between 0.77 and 0.91, reaching a significant level (*ps* < 0.001) (Table 2). This indicates that all 12 items had good psychometric properties. Meanwhile, the critical ratio (CR) method was also employed to determine the level of item discrimination for each factor of the Ch-MCOI. This method typically sets 27% of each factor’s total score as the boundary point. The factors with a score in the top 27% were classified as a high-score group (n = 151), and those with a score in the bottom 27% were classified as a low-score group (n = 151). Then, an independent sample t-test was adopted to test whether each item could achieve a significant level. Our results revealed that Ch-MCOI CRs were between 18.44 and 33.46, reaching a significant level (*ps* < 0.001). This suggests that Ch-MCOI item discrimination is high.

**Table 2:** Analysis of the items of the Chinese version of the Multidimensional Competitive Orientation Inventory.

Item	ITC	CR
Item 1	0.82**	27.41**
Item 2	0.84**	28.62**
Item 3	0.79**	25.75**
Item 4	0.84**	29.18**
Item 5	0.85**	30.99**
Item 6	0.77**	18.44**
Item 7	0.83**	28.35**
Item 8	0.82**	20.59**
Item 9	0.85**	30.65**
Item 10	0.84**	24.94**
Item 11	0.90**	31.99**
Item 12	0.91**	33.46**

*ITC* item-total correlation, *CR* critical ratio.

**p < 0.01, *p < 0.05.

### Construct validity

Our ESEM analysis on Subsample 1 demonstrated a four-factor structure for the Ch-MCOI, which provides a satisfactory fit to the data (χ^2^ = 68.33, df = 24, *p* < 0.01; CFI = 0.98; TLI = 0.94; RMSEA = 0.06 [90% CI 0.042, 0.074]; SRMR = 0.02). The results showed strong target loadings (|λ|= 0.55–0.89, M = 0.72) with minimal cross-loading (|λ|= 0.00–0.28, M = 0.06) (Table 3). Moreover, there was a significant correlation across all four factors (r = -0.17 ~ 0.48, *p* < 0.01), consistent with the original research^27^. Given that this result supports the proposed four-factor structure of the English version of the MCOI^27^, we used the same terms to name these four factors: “Hyper-Competitive Orientation (HCO)”, “Self-Developmental Competitive Orientation (SDCO)”, “Anxiety-Driven Competition Avoidance (ADCA)”, and “Lack of Interest in Competition (LIC)”.

**Table 3:** Results of the exploratory structural equation modeling on the Chinese version of the Multidimensional Competitive Orientation Inventory.

Items	Lack of interest toward competition	Hyper-competitive orientation	Anxiety-driven competition avoidance	Self-developmental competitive orientation
(1) I rarely feel motivated to compete with somebody	**0.72**	*−0.02*	*0.03*	*−0.02*
(2) There is always something I’d rather do than taking part in a competitive situation	**0.71**	*−0.01*	0.15	*0.01*
(3) I don’t care about competitions	**0.66**	*0.00*	*−0.05*	*0.06*
(4) The most important is winning, no matter what	*−0.02*	**0.75**	*0.06*	*0.00*
(5) I am willing to do whatever it takes to win	*−0.04*	**0.75**	*−0.01*	*0.06*
(6) I will do anything to win, even nasty things	*0.13*	**0.62**	*−0.02*	*0.01*
(7) I feel distressed in a competitive environment, so I avoid them whenever I can	0.28	0.13	**0.55**	*−*0.09
(8) I feel pressured in competitive situations	*−0.02*	*−*0.11	**0.81**	0.15
(9) Even the smallest competition makes me feel anxious	*0.06*	*0.04*	**0.69**	*−0.01*
(10) Competitive situations allow me to bring the best out of myself	*−0.05*	*0.05*	0.13	**0.71**
(11) I enjoy testing myself in competitive situations	0.06	*0.02*	*−0.05*	**0.85**
(12) I enjoy competition as it allows me to discover my abilities	*−0.01*	*−0.01*	*−0.01*	**0.89**

All factor loadings are standardized. Loadings in bold represent the final items and all are statistically significant at *p* < 0.001. Non-significant cross-loadings are in italics.

**Figure 1:**
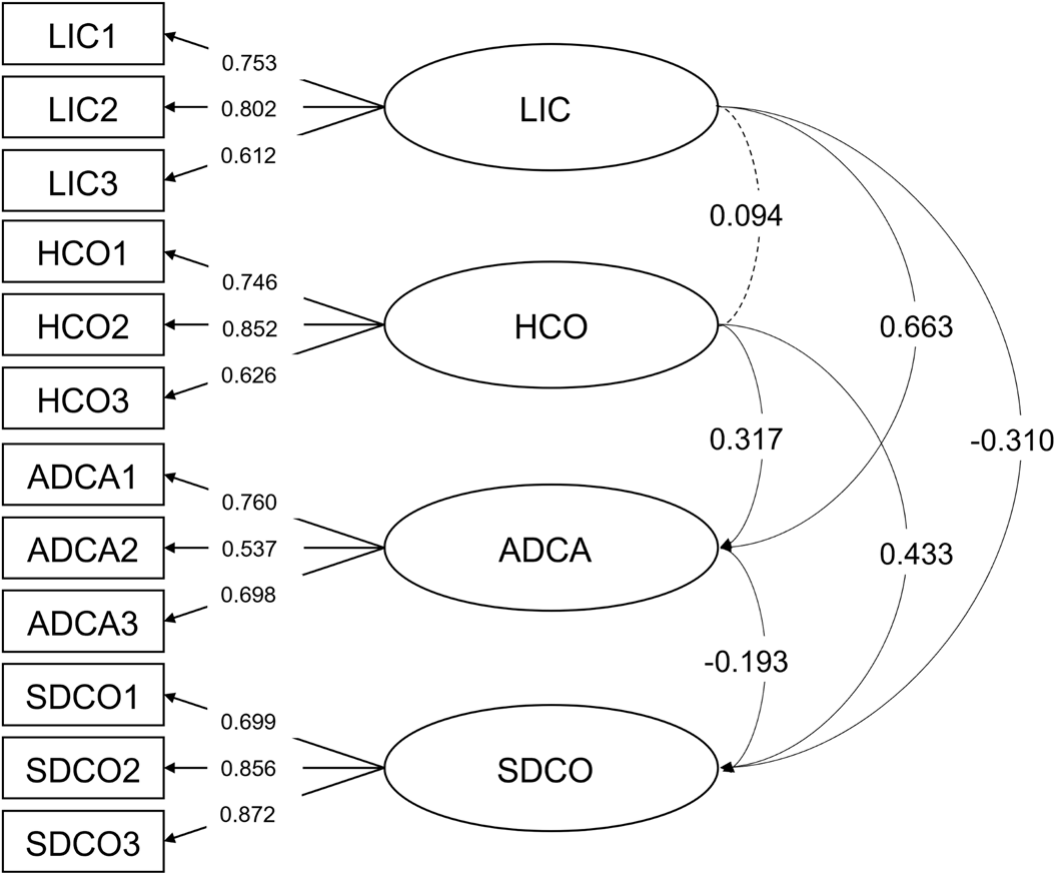
The four-factor confirmatory factor analysis model of the Chinese version of the Multidimensional Competitive Orientation Inventory for the validation subsample (n = 561). Each number alongside the lines indicates standardized factor loading. *LIC* lack of interest in competition, *HCO* hyper-competitive orientation, *ADCA* anxiety-driven competition avoidance, *SDCO* self-developmental competitive orientation.

Subsequently, to verify the factor structure identified, we carried out a confirmatory factor analysis (CFA) on Subsample 2. Our results showed that the model fit indices were acceptable (χ^2^ = 184.05, df = 48, CFI = 0.95, TLI = 0.93, SRMR = 0.05, RMSEA = 0.07, NFI = 0.93, IFI = 0.95) (Table 4**, **Fig. 1). This solution confirmed that the four-factor structure of the Ch-MCOI is adequate.

**Table 4:** Goodness-of-fit indices for the Chinese version of the Multidimensional Competitive Orientation Inventory.

χ^2^	df	CFI	TLI	SRMR	RMSEA	NFI	IFI
184.05	48	0.95	0.93	0.05	0.07	0.93	0.95

*χ*^*2*^ Chi-square, *df* degrees of freedom, *CFI* comparative fit index, *TLI* Tucker–Lewis Index, *SRMR* standardized root mean square residual, *RMSEA* root-mean-square error of approximation, *NFI* Bentler–Bonett normed fit index, *IFI* incremental fit index.

### Reliability

To assess the reliability of the CH-MCOI, Cronbach’s α and composite reliability (CR) were calculated for each factor of the Ch-MCOI. The Cronbach’s α for each of the four factors was acceptable, between 0.75 and 0.85 (Table 5). Similarly, the CR for each of the four factors was also acceptable, between 0.81 to 0.90 (Table 5). These results demonstrate the satisfactory internal consistency of the Ch-MCOI. The ICC was used to assess the 2-week test–retest reliability. The ICC coefficient for each of the four factors was between 0.67 and 0.73 ( *p* < 0.001), which was significant (*ps* < 0.01) (Table 5**)**. The results indicated an adequate test–retest reliability for the Ch-MCOI.

**Table 5:** Internal consistency and test–retest reliability of each factor of the Chinese version of the Multidimensional Competitive Orientation Inventory.

Scales	Test/retest			CR	ICC
	Cronbach's α	*M*	*SD*		
Lack of interest toward competition	0.76/0.73	3.20/3.24	1.02/1.02	0.83	0.69**
Hyper-competitive orientation	0.77/0.74	3.00/2.81	1.02/1.02	0.84	0.68**
Anxiety-driven competition avoidance	0.75/0.71	3.62/3.63	0.95/0.92	0.81	0.67**
Self-developmental competitive orientation	0.85/0.86	4.00/4.07	1.11/1.08	0.90	0.73**

*M* mean, *SD* standard deviation, *CR* composite reliability, *ICC* intraclass correlation.

**p < 0.01, *p < 0.05.

### Convergent and concurrent validity

The Average Variance Extracted (AVE) values corresponding to the HCO, SDCO, ADAC, and LIC are 0.64, 0.74, 0.60, and 0.63 respectively. As stated before, AVE ≥ 0.5 confirms the convergent validity. The convergent validity of the Ch-MCOI is established since all the AVE values of the four factors are greater than 0.50.

Regarding the concurrent validity of the Ch-MCOI, we found that both the Ch-CD-RISC and Ch-WEMWBS were positively correlated with the Ch-MCOI’s SDCO subscale, whereas both the Ch-CD-RISC and Ch-WEMWBS were negatively correlated with the Ch-MCOI’s ADCA. Furthermore, both the Ch-APS discrepancy subscale and high standards subscale were positively correlated with both the Ch-MCOI’s HCO subscale and ADCA subscale, while both the Ch-APS high standards subscale and order subscale were positively correlated with the Ch-MCOI’s SDCO subscale. In addition, the Ch-APS high standards subscale was negatively correlated with the Ch-MCOI’s LIC subscale. Finally, all five Ch-MPS-f subscales were positively correlated with both the Ch-MCOI’s HCO subscale and SDCO subscale, whereas the four Ch-MPS-f subscales were positively correlated with both the ADCA subscale of the Ch-MCOI. However, the organization subscale was negatively correlated with the Ch-MCOI’s LIC subscale. The Ch-CAS benign competitive attitude subscale was positively correlated with the Ch-MCOI’s SDCO subscale, while the Ch-CAS negative competitive attitude subscale correlated positively with the Ch-MCOI’s HCO subscale (Table 6).

**Table 6:** Partial correlations between each factor of the Chinese version of the Multidimensional Competitive Orientation Inventory and other assessments.

Scale	Lack of interest toward competition	Hyper-competitive orientation	Anxiety-driven competition avoidance	Self-developmental competitive orientation
Connor-Davidson Resilience Scale	0.01	−0.05	−0.11**	0.37**
Warwick-Edinburgh Mental Well-being Scale	0.02	−0.03	−0.18**	0.39**
Almost Perfect Scale—discrepancy	0.03	0.22**	0.23**	0.05
Almost Perfect Scale—high standards	−0.18**	0.12**	0.16**	0.32**
Almost Perfect Scale—order	−0.02	−0.01	0.05	0.32**
Frost Multidimensional Perfectionism Scale—concern over mistakes	0.03	0.15**	0.14**	0.14**
Frost Multidimensional Perfectionism Scale—parental criticism	−0.03	0.14**	0.08**	0.27**
Frost Multidimensional Perfectionism Scale—personal standards	−0.05	0.14**	0.13**	0.20**
Frost Multidimensional Perfectionism Scale—doubts about actions	−0.01	0.07*	−0.02	0.32**
Frost Multidimensional Perfectionism Scale—organization	−0.12**	0.19**	0.08*	0.24**
Competition Attitude Scale—benign	–	–	–	0.62**
Competition Attitude Scale—negative	–	0.48**	–	–

**p < 0.01, *p < 0.05.

To summarize, the correlation tendency between the four subscales and these measurements was similar to that reported by Orosz et al.^27^, verifying that the Ch-MCOI had good concurrent validity.

### Gender effects

Before evaluating whether there is a gender effect on the Ch-MCOI scores, we conducted invariance tests to check for equivalence across genders. Participants were split into a male group (N = 567) and a female group (N = 554). As shown in Table 7, a moderate model fit was obtained for both groups (CFI ≥ 0.92, RMSEA ≤ 0.09, SRMR ≤ 0.06), following the suggestion by^46^. Then, the test for the configural invariance (M0) supported a moderate fit to the data across gender groups (CFI = 0.94, RMSEA = 0.08, SRMR = 0.06), which can act as a baseline model to compare subsequent models^44^. Next, we assessed metric invariance (M1) by constraining all factor loadings to be equal across gender groups. The metric invariance could be assumed across gender groups as evidenced by a nonsignificant drop in model fit (ΔCFI = 0.01, ΔRMSEA = 0.00, ΔSRMR = 0.00), according to practical fit indices: ΔCFI, ΔRMSEA, and ΔSRMR are within the threshold of ≤ 0.01^47,48^. Finally, we evaluated scalar invariance (M2) by constraining the item intercepts to be equal across gender groups. The scalar invariance could also be assumed across gender groups as evidenced by a nonsignificant drop in model fit (ΔCFI = 0.01, ΔRMSEA = 0.00, ΔSRMR = 0.00). Therefore, these results indicate that the factor structure, factor loadings, and intercepts of the Ch-MCOI were invariant across gender groups.

**Table 7:** Test of measurement invariance of the four-factor model of the Chinese version of the Multidimensional Competitive Orientation Inventory across gender.

Models	*χ*^2^(ML)	*df*	*p*	CFI	TLI	RMSEA	SRMR	$\Delta$CFI	$\Delta$RMSEA	$\Delta$SRMR
Male (N = 567)	171.63	48	< 0.00	0.95	0.93	0.07	0.05	–	–	–
Female (N = 554)	246.27	48	< 0.00	0.92	0.89	0.09	0.06	–	–	–
Configural (M0)	417.90	96	< 0.00	0.94	0.91	0.08	0.06	–	–	–
Metric (M1)	435.56	104	< 0.00	0.93	0.92	0.08	0.06	0.01	0.00	0.00
Scalar (M2)	519.03	112	< 0.00	0.92	0.90	0.08	0.06	0.01	0.00	0.00

*χ*^*2*^(*ML*) maximum likelihood Chi-square, *df* degrees of freedom, *CFI* comparative fit index, *TLI* Tucker–Lewis Index, *RMSEA* root-mean-square error of approximation, *SRMR* standardized root mean square residual, $\Delta$*CFI* difference in comparative fit index, $\Delta$*RMSEA* difference in root-mean-square error of approximation, $\Delta$*SRMR* difference in standardized root mean square residual.

Based on the results described above, we further performed the independent sample t-test to assess how gender affects scores on the Ch-MCOI. Our results revealed that Chinese males achieved a significantly higher mean score on both the HCO subscale (*t*_(1074)_ = 8.03, *p* < 0.001) and the SDCO subscale (*t*_(1119)_ = 7.68, *p* < 0.001) than Chinese females. In contrast, Chinese females achieved a significantly higher mean score on the ADCA subscale than Chinese males (*t*_(1113)_ = 5.58, *p* < 0.001) **(**Table 8**).**

**Table 8:** Gender differences in the Chinese version of the Multidimensional Competitive Orientation Inventory.

The four subscales	Male (n = 567) (*M* ± *SD*)	Female (n = 554) (*M* ± *SD*)	Comparison
Lack of interest toward competition	3.23 ± 1.13	3.17 ± 1.00	*t* (1106) = 0.80
Hyper-competitive orientation	3.05 ± 1.17	2.54 ± 0.93	*t* (1073) = 8.03**
Anxiety-driven competition avoidance	3.46 ± 1.06	3.80 ± 0.96	*t* (1113) = −5.58**
Self-developmental competitive orientation	4.00 ± 1.12	3.51 ± 1.00	*t* (1119) = 7.69**

*M* mean; *SD* standard deviation.

**p < 0.01, *p < 0.05.

## Discussion

This study clearly demonstrated that the Ch-MCOI has appropriate psychometric properties. Specifically, it has solid convergent validity, strong internal consistency, and temporal stability. Each factor of the Ch-MCOI demonstrates a meaningful relationship pattern with mental well-being, resilience, and perfectionism. In comparison with the English version of the MCOI, the Ch-MCOI achieved similar results for resilience, except for the lack of interest in competition.

The Ch-MCOI, similar to the original English version, could replace conventional monistic or dualistic measurements of competition^49,50^ as the LIC subscale had a significant negative correlation with both a positive and negative competitive attitude^21^. The HCO subscale only had a positive correlation with a negative competitive attitude. The ADCA subscale had a negative relationship with a positive competitive attitude and a positive relationship with a negative competitive attitude. Conversely, the SDCO subscale had a positive correlation with a benign competitive attitude and a negative correlation with a negative competitive attitude. It shows the four different facets of competition have a diverse pattern with the negative and positive aspects of competition and it underlies the necessity of using multidimensional competitive measurement tools. In addition, regarding the Chinese sample, the LIC could not predict resilience. The SDCO was positively related to almost all factors of perfectionism, in line with prior studies^9,51^. The HCO was also related to the most negative aspect of Perfectionism. The LIC was negatively related to High Standards subscale, which is different from the original version, and had no relationship with Perfectionism characteristics, in line with expectations.

Compared to the original version, Chinese respondents scored relatively high on the LIC and ADCA subscales and relatively low on the SDCO subscale. Surprisingly, Chinese participants scored higher than Hungarians on the HCO subscale. One of the reasons for this could be the competitive societal environment in China. As a consequence of the rise of the socialist market economy and the burgeoning of the Chinese population, young Chinese confront intense competition today. They must engage in *neijuan* (irrational competition) with others, but they must also win or they could easily lose their jobs^24^. Due to the pressure to compete with others, they are unable to enjoy competition. This might be related to the combination of relatively low self-developmental competitive orientation and strong hyper-competitive pressures. Meanwhile, the LIC scores were also inversely associated with High Standards of Perfectionism scores. This link was not significant in the Hungarian sample. This result might be consistent with the Taoist philosophy: *“When there is no desire, all things are at peace”* (LaFargue, 1994). To summarize, it appears that a growing percentage of young people are exhausted by competition and want to “Lie Flat”. Similarly, scores of the ADCA in the Chinese sample were inversely correlated with well-being and positively correlated with maladaptive Perfectionism. It may be inspired by Confucianism, which holds that those who like competition are not *Junzi*^20^ and may be under more moral strain. This is in line with previous studies showing that all Junzi characteristics are adversely correlated with hyper-competitiveness (Ge et al.^20^. It is possible to interpret hyper-competitiveness as a hallmark of *xiaoren* (which can be translated as “villain”), which is the opposite of *Junzi*. This can create an internal conflict among young Chinese adults who live in an extremely competitive social environment and who are trying to do so without becoming *xiaoren*, even if they have a strong desire to win.

Finally, the Ch-MCOI showed gender differences that were not observed in the original version. Males scored lower on the LIC subscale, HCO subscale, and the SDCO subscale, while females scored significantly higher on the ADCA subscale. These results are consistent with Confucian values. According to these values, women are discouraged from engaging in any type of competition or from being aggressive^52^. Even if they choose to compete, females feel much more moral pressure than men.

### Strengths and limitations

To the best of our knowledge, this is the first study to examine the psychometric properties of the MCOI in the Chinese-speaking population. Another strength of this study is relatively the large sample of adults with an age range from 18 to 63 years old. However, the present study may potentially be limited by a questionable generalization of our results to the wider, general Chinese population in other regions such as Hongkong and Taiwan.

Moreover, other forms of reliability and validity tests could have been employed to strengthen the evidence of its applicability to the Chinese-speaking population. This obviously motivates future research clarifying this issue.

## Conclusion

In terms of factor structure, internal consistency, temporal reliability, and convergent validity, the Ch-MCOI seems to have suitable psychometric features. The results of the LIC and ADCA subscales may reflect cultural values as well as Taoism and Confucianism principles. Thus, it is a valid instrument for the evaluation of the different aspects of competition in the Chinese-speaking population. Future research could examine individual variations in competitiveness in educational, occupational, and health-related situations by using this measurement in the Chinese cultural context. In addition, researchers could also use this tool in combination with neuroscience to find neural processes associated with different competition facets.

## Data Availability

The datasets used and/or analysed during the current study available from the corresponding author on reasonable request.

## References

[1] Horney K (1937). Neurotic Personality of Our Time.

[2] Deutsch M (1949). A theory of co-operation and competition. Hum. Relat..

[3] Deutsch M (1949). An experimental study of the effects of co-operation and competition upon group process. Hum. Relat..

[4] Kc RP, Kunter M, Mak V (2018). The influence of a competition on noncompetitors. Proc. Natl. Acad. Sci..

[5] Pisauro MA, Fouragnan E, Arabadzhiyska DH, Apps MAJ, Philiastides MG (2022). Neural implementation of computational mechanisms underlying the continuous trade-off between cooperation and competition. Nat. Commun..

[6] Armstrong, J. Review of Alfie Kohn, no contest: The case against competition. *J. Market.* (1988).

[7] Arrindell WA, Steptoe A, Wardle J (2003). Higher levels of state depression in masculine than in feminine nations. Behav. Res. Ther..

[8] Gilbert, P. *Compassion: Conceptualisations, Research and Use in Psychotherapy.* 9–74 (Routledge, 2005).

[9] Ryckman RM, Hammer M, Kaczor LM, Gold JA (1996). Construction of a personal development competitive attitude scale. J. Pers. Assess..

[10] Orosz G (2018). The four faces of competition: The development of the multidimensional competitive orientation inventory. Front. Psychol..

[11] Ryckman RM, Hammer M, Kaczor LM, Gold JA (1990). Construction of a hypercompetitive attitude scale. J. Pers. Assess..

[12] Ryckman RM, Thornton B, Gold JA (2009). Assessing competition avoidance as a basic personality dimension. J. Psychol..

[13] Albouza, Y., Wach, M. & Chazaud, P. Factorial validation and measurement invariance of the French version of the multidimensional competitive orientation inventory (FVMCOI) in the sport field. *Prat. Psychol.* (2020).

[14] Fülöp, M. & Orosz, G. *State of the Art in Competition Research*. 10.1002/9781118900772.etrds0317 (2015).

[15] Jamaluddin SF, Adi SP, Lufityanto G (2020). Social influences on cheating in collectivistic culture: Collaboration but not competition. Group Dyn. Theory Res. Pract..

[16] Triandis, H. C., Collectivism v. Individualism: A Reconceptualisation of a Basic Concept in Crosscultural Social Psychology. (1988).

[17] Liu SS, Shteynberg G, Morris MW, Yang Q, Galinsky AD (2020). How does collectivism affect social interactions? A test of two competing accounts. Pers. Soc. Psychol. Bull..

[18] Brindley E (2009). "Why use an ox-cleaver to carve a chicken?" The sociology of the Junzi ideal in the Lunyu. Philos. East West.

[19] Cua AS (2007). Virtues of Junzi. J. Chin. Philos..

[20] Ge X, Li X, Hou Y (2021). Confucian ideal personality traits (Junzi personality): Exploration of psychological measurement. Acta Psychol. Sin..

[21] Chen GP, Li J, Lu F (2003). The Chinese version of competition attitude scale. J. Psychol. Sci..

[22] Young IF (2021). A multidimensional approach to the relationship between individualism-collectivism and guilt and shame. Emotion.

[23] Yi D (2022). Does involution cause anxiety? An empirical study from Chinese universities. Int. J. Environ. Res. Public Health.

[24] Dou G, Li G, Yuan Y, Liu B, Yang L (2022). Structural dimension exploration and measurement scale development of employee involution in China’s workplace field. Int. J. Environ. Res. Public Health.

[25] Xie X, Yu Y, Chen X, Chen X (2006). The measurement of cooperative and competitive personality. Acta Psychol. Sin..

[26] Niederle M, Vesterlund L (2011). Gender and competition. Annu. Rev. Econ..

[27] Orosz G (2018). The four faces of competition: The development of the Multidimensional Competitive Orientation Inventory. Front. Psychol..

[28] Beaton DE, Bombardier C, Guillemin F, Ferraz MB (2000). Guidelines for the process of cross-cultural adaptation of self-report measures. Spine.

[29] Li YL, Tan LM, Liu JJ, Lou FL (2013). Probe into intermediary role of general self-efficacy of college students in relationship between emotional creativity and mental flexibility. Chin. Nurs. Res..

[30] Liu YC, Guo LN, Liu K (2016). Validity and reliability of Warwick-Edinburgh Mental Well-being Scale (WEMWBS) in older people. Chin. Ment. Health J..

[31] Yang L, Liang BY, Zhang XG, Wu YC (2007). The Chinese version of Almost Perfect Scale-Revised. Stud. Psychol. Behav..

[32] Cheng SK, Chong GH, Wong CW (1999). Chinese Frost Multidimensional Perfectionism Scale: A validation and prediction of self-esteem and psychological distress. J. Clin. Psychol..

[33] Smith MD (1996). Structural equation modeling: Concepts, issues, and applications. Statistician.

[34] West, S. G., Finch, J. F., & Curran, P. J., Structural equation models with non-normal variables: Problems and remedies. (1995).

[35] Marsh HW (2009). Exploratory structural equation modeling, integrating CFA and EFA: Application to students' evaluations of university teaching. Struct. Equ. Model. Multidiscip. J..

[36] Morin, A. J. S., Marsh, H. W., and Nagengast, B. . *Structural Equation Modeling: A Second Course* (eds. Hancock, G. R., R. O. Mueller Inc.). 395–436 (Information Age Publishing, 2013).

[37] Tóth-Király I, Morin AJS, Bőthe B, Orosz G, Rigó A (2017). Investigating the multidimensionality of need fulfillment: A bifactor exploratory structural equation modeling representation. Struct. Equ. Model. Multidiscip. J..

[38] Taasoobshirazi G, Wang S (2016). The performance of the SRMR, RMSEA, CFI, and TLI: An examination of sample size, path size, and degrees of freedom. J. Appl. Quant. Methods.

[39] Brown TA (2015). Confirmatory Factor Analysis for Applied Research.

[40] Raykov T (1997). Estimation of composite reliability for congeneric measures. Appl. Psychol. Meas..

[41] Cronbach LJ (1951). Coefficient alpha and the internal structure of tests. Psychometrika.

[42] Niederle M, Vesterlund L (2008). Gender differences in competition. Negotiat. JX..

[43] Gneezy U, Rustichini A (2004). Gender and competition at a young age. Am. Econ. Rev..

[44] van de Schoot R, Lugtig P, Hox J (2012). A checklist for testing measurement invariance. Eur. J. Dev. Psychol..

[45] Vandenberg RJ, Lance CE (2000). A review and synthesis of the measurement invariance literature: Suggestions, practices, and recommendations for organizational research. Organ. Res. Methods.

[46] Schermelleh-Engel K, Moosbrugger H, Müller H (2003). Evaluating the fit of structural equation models: Tests of significance and descriptive goodness-of-fit measures. Methods Psychol. Res..

[47] Chen FF (2007). Sensitivity of goodness of fit indexes to lack of measurement invariance. Struct. Equ. Model. Multidiscip. J..

[48] Cheung GW, Rensvold RB (2002). Evaluating goodness-of-fit indexes for testing measurement invariance. Struct. Equ. Model. Multidiscip. J..

[49] Cen Y (2009). A study on construction and psychometric properties of competitiveness scale of college students. Psychol. Res..

[50] Zhou X, Zheng X, Yan B (2007). Revision of competition scale for college students in middle school students. J. South China Normal Univ..

[51] Thornton B, Ryckman RM, Gold JA (2011). Competitive orientations and the type a behavior pattern. Psychology.

[52] Patt-Shamir G (2010). The value in storytelling: Women’s life-stories in Confucianism and Judaism. Dao.

